# Polymorphic Transformation and Magnetic Properties of Rapidly Solidified Fe_26.7_Co_26.7_Ni_26.7_Si_8.9_B_11.0_ High-Entropy Alloys

**DOI:** 10.3390/ma12040590

**Published:** 2019-02-15

**Authors:** Zequn Zhang, Kaikai Song, Ran Li, Qisen Xue, Shuang Wu, Delong Yan, Xuelian Li, Bo Song, Baran Sarac, Jeong Tae Kim, Parthiban Ramasamy, Li Wang, Jürgen Eckert

**Affiliations:** 1 School of Mechanical, Electrical & Information Engineering, Shandong University (Weihai), Weihai 264209, China; zequnzhang6018@hotmail.com (Z.Z.); xqs_sky@163.com (Q.X.); muluoyannanduxi@163.com (S.W.); yandelong2@yeah.net (D.Y.); hunterlxl@163.com (X.L.); wanglihxf@sdu.edu.cn (L.W.); 2 School of Materials Science and Engineering, Beihang University, Beijing 100191, China; liran@buaa.edu.cn; 3 College of Marine Science, Shandong University (Weihai), Weihai 264209, China; songbo828615@aliyun.com; 4 Erich Schmid Institute of Materials Science, Austrian Academy of Sciences, A-8700 Leoben, Austria; baran.sarac@oeaw.ac.at (B.S.); jeongtae.kim@oeaw.ac.at (J.T.K.); parthiban.ramasamy@oeaw.ac.at (P.R.); juergen.eckert@unileoben.ac.at (J.E.); 5 Department Materials Physics, Montanuniversität Leoben, A-8700 Leoben, Austria

**Keywords:** amorphous alloys, high-entropy alloys, polymorphic transformation, magnetic properties

## Abstract

In this work, the microstructural evolution and magnetic performance of the melt-spun amorphous and amorphous-crystalline Fe_26.7_Co_26.7_Ni_26.7_Si_8.9_B_11.0_ high-entropy alloys (HEAs) during crystallization were investigated, respectively. Upon heating fully amorphous ribbons, a metastable BCC supersaturated solid solution together with a little Ni_31_Si_12_ crystals first precipitated and then the (Fe,Co)_2_B crystals formed until the full crystallization was achieved. With further increasing temperature after full crystallization, a polymorphic transformation from a metastable BCC phase to two types of FCC solid solutions occurred. For the amorphous-crystalline HEAs, the dominant crystallization products were the metastable FCC but not BCC crystals. During crystallization, the primary metastable FCC crystals first transform into the metastable BCC crystals and then the newly-generated BCC phase transforms into two types of FCC phases with further increasing temperature. This temperature dependence of the gradual polymorphic transformation results in the change of magnetic properties of the present high-entropy amorphous alloys.

## 1. Introduction

A novel paradigm for alloy design was proposed based on the mixing of multiple elements in an equimolar or near-equimolar composition [1,2]. These novel kinds of alloys usually contain five or more principal elements while each principal element should have a concentration between 5 and 35 at % [1,2]. Since their liquid or random solid solution states possess significantly higher mixing entropies than conventional alloys, the designed multicomponent alloys were named as high-entropy alloys (HEAs) [1,2]. Their important microstructural characteristics are that the high configurational entropy of the random mixing of elements usually facilitates the formation of solid solution phases with simple structures [3,4,5,6,7,8] such as face-centered-cubic (FCC), body-centered cubic (BCC), hexagonal close-packed structure (HCP), or a mixture of them [3,4,5,6,7,8,9,10,11,12,13,14,15]. Even in some cases, a little intermetallic compounds precipitate in the single solid solution matrix, leading to the further enhancement of the mechanical properties of HEAs [3,5]. Additionally, recent studies have shown that the HEAs fabricated using different methods exhibit superior mechanical and physical properties such as high hardness, ultrahigh fracture toughness, good wear resistance, good tribological properties, excellent high temperature softening resistance, good magnetic properties, and good corrosion resistance [3,4,5,6,7,8,9,16,17,18,19,20,21,22,23,24]. It has been claimed that these promising properties are ascribed to the high-entropy of alloys, the sluggish diffusion, the cocktail effect, and the large lattice distortion of the respective structures [3,4,5,6,7,8,9,12,25,26,27]. 

Until now, more than three hundred reported HEAs have been designed based on more than thirty elements [5]. In order to increase the specific strength and further explore the soft magnetic properties of HEAs, elements like B, Si, P, and/or C with relatively small atomic weights are introduced into FeCoNi medium entropy alloys [28,29,30,31]. Surprisingly, some of these types of HEAs are able to easily form metallic glasses [28,29,30,31]. Then the high-entropy glass-forming composites could be an excellent candidate to fabricate metallic glass composites since the dominant crystallization products are single FCC solid solution for some cases [32,33]. However, by the traditional rapid solidification, it is difficult to control the precipitation of crystals in the glassy matrix due to the precipitation of brittle intermetallic compounds and the grain coarsening [34]. Recently, a flash annealing method was used to fabricate ductile metallic glass composites especially in CuZr-based glass-forming systems [35,36,37]. The fabricated metallic glass composites show not only excellent mechanical properties but also good magnetic properties (i.e., Fe-based alloys) [38]. However, it is not feasible for most glass-forming compositions since the dominant high-temperature precipitates in the glassy matrix during flash annealing are intermetallic compounds but not ductile crystalline phase. Therefore, it is necessary to explore high-entropy glass-forming compositions, which satisfy the requirements for flash annealing and meanwhile investigate their crystallization behaviors. Recently, Li et al. found that an FCC solid solution in an amorphous matrix transforms into a BCC phase in the case of the crystalline-amorphous Fe_26.7_Co_26.7_Ni_26.6_Si_9_B_11_ HEAs, while a BCC phase transforms into an FCC phase and then into a BCC phase by gradually heating the amorphous Fe_26.7_Co_28.5_Ni_28.5_Si_4.6_B_8.7_P_3_ HEAs [32,33]. As a result, the magnetic properties of such alloys can be effectively tailored by adjusting their phase structures upon crystallization. However, Zhang et al. demonstrated that by applying appropriate heat treatments on Fe_26.7_Co_26.7_Ni_26.7_Si_8.9_B_11.0_ HEAs, only the dominant structural framework gradually transformed from eutectic structures to FCC dendrites and ultimately the (Fe,Co)_2_B crystals became isolated as dominant reinforcement particles distributed in the interdendritic regions [39]. No obvious polymorphic phase transformation could be observed under these (quasi) equilibrium conditions. Therefore, it was reasonable to infer that the observed polymorphic transformation should occur under non-equilibrium conditions [32,33]. In order to clearly understand such solid phase transformations and their influence on the magnetic properties in details, more investigations are required.

In this work, both the fully amorphous and crystalline-amorphous Fe_26.7_Co_26.7_Ni_26.7_Si_8.9_B_11.0_ HEAs were fabricated by rapid solidification. Their crystallization sequences upon heating were investigated to further clarify the polymorphic transformation between both FCC and BCC phases. Furthermore, the effect of these phase transformations on their magnetic properties were also studied.

## 2. Materials and Methods 

Ingots with a nominal composition of Fe_26.7_Co_26.7_Ni_26.7_Si_8.9_B_11.0_ were fabricated by arc-melting appropriate amounts of the constituting elements (Fe, Co, Ni, and Si, >99.9% purity) and the Fe_45.32_B_54.68_ master alloy (>99.9% purity) under a Ti-gettered argon atmosphere. In order to guarantee chemical homogeneity, the master ingots were remelted at least four times by arc-melting and then were purified by fluxing with B_2_O_3_ using a high-frequency induction furnace under an argon atmosphere. Afterwards, the ribbons were fabricated by melt-spinning under an argon atmosphere. By decreasing the rotation speed of the copper wheel during melt spinning from about 32.26 m/s to 11.52 m/s, both full amorphous ribbons (AR) and amorphous-crystalline ribbons (ACR) were fabricated, respectively. Thermal properties were examined by three differential scanning calorimetry (DSC, Mettler Toledo TG/DSC 1, Zurich, Switzerland, Perkin Elmer 8500, Waltham, MA, USA and Netzsch 404, Selb, Germany) devices at a heating rate of 20 K/min. The phase analysis of the as-quenched alloys was carried out by X-ray diffraction (XRD, Rigaku D/max-rB, Cu *K_α_* radiation, Tokyo, Japan). During XRD measurements, the graphite monochromator was used to filter out Cu *K_β_* X-rays. In order to avoid effects from Cu-K*_α1_*_/_*_α2_* X-rays, the XRD measurements were repeated as least two times for all samples. Furthermore, the transmission electron microscope (TEM, JEOL-2100, Tokyo, Japan) was also adopted to further identify the phase formation. The samples for the TEM measurements were prepared by a dual Focused Ion Beam System (FIB, HELIOS NanoLab 600i, Hillsboro, OR, USA) set up in a scanning electron microscopy (SEM, FEI Sirion, Hillsboro, OR, USA). Magnetic properties (i.e., saturation magnetization *B_s_* and coercive force *H_c_*) were measured with a vibrating sample magnetometer (VSM, Lakeshore 7407, Westerville, USA and SQUID-VSM, Quantum Design, San Diego, CA, USA) under fields of 800 or 1500 kA/m and a DC *B*–*H* loop tracer (MATS-2010SD, Loudi, China), respectively.

## 3. Results and Discussion

### 3.1. Microstructural Evolution of the Amorphous High-Entropy Alloy during Heating

**Figure 1:**
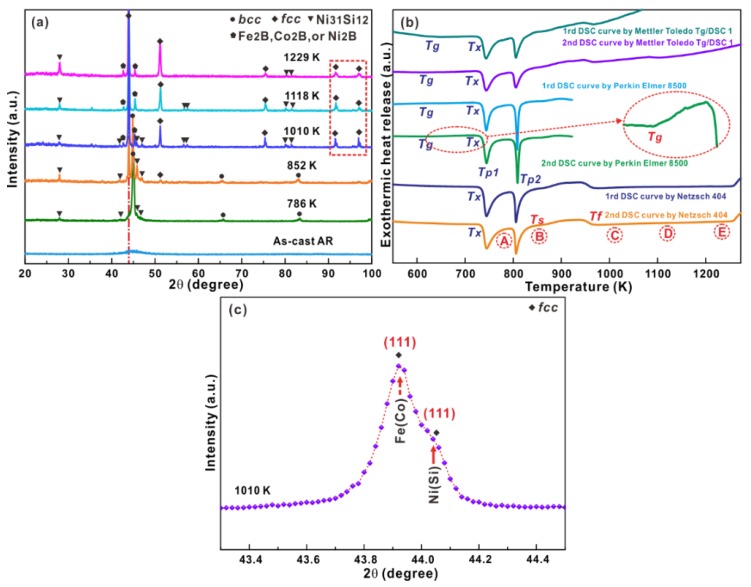
(**a**) X-ray diffraction patterns of the melt-spun AR ribbons and samples heated to different temperatures, (**b**) the DSC curve of the melt-spun AR ribbons measured by different DSC devices (inset: the glass transition zone), (**c**) the diffraction peaks of two type of FCC phases along the zone axis (111) for the AR sample heated to 1010 K, which were taken from Figure 1a.

Figure 1a shows the XRD patterns for the melt-spun AR samples and samples annealed at different temperatures at the heating and cooling rate of 20 K/min. Only broad diffuse diffraction maxima without crystalline peaks could be observed for the melt-spun AR ribbons fabricated at a wheel speed of about 32.26 m/s, revealing their amorphous nature. As shown in Figure 1b, the DSC curve of the AR samples exhibited a glass transition followed by two exothermic peaks related to crystallization upon heating. After full crystallization, it seemed that broad endothermic reactions appeared after crystallization (i.e., point B in Figure 1b) and before melting, implying that some solid phase transformations could happen. Different types of DSC devices were also adopted on the AR samples several times to confirm the authenticity of the endothermic reactions. Obviously, some solid phase transformations after crystallization of the amorphous AR samples indeed exist. The glass transition temperature (*T_g_*) was measured as 623.6 ± 9.1 K, while the onset temperature (*T_x_*), the first peak temperature (*T_p1_*), and the second temperature (*T_p2_*) of crystallization were determined to be 731.8 ± 3.1 K, 743.3 ± 1.2 K, and 805.6 ± 3.5 K, respectively. After crystallization, the onset (*T_s_*) and final temperatures (*T_f_*) of the endothermic reactions were measured to be 862.0 ± 4.0 K and 966.0 ± 4.1 K, respectively, while the corresponding released endothermic enthalpy was 38.6 ± 4.6 J/g.

In order to clarify the microstructural evolutions during the whole crystallization, XRD measurements were performed for samples heated to different temperatures (i.e., A: 786 K, B: 852 K, C: 1010 K, D: 1118K, and E: 1229 K). The crystalline volume fractions for the samples after annealing at different temperatures could be determined to be 55.6 ± 0.5 vol % (A), 99.9 ± 0.1 vol % (B), 100 vol % (C), 100 vol % (D), and 100 vol % (E), respectively, according to the temperature dependence of exothermic enthalpy during crystallization. The corresponding XRD patterns displayed in Figure 1a revealed the formation of a BCC phase together with a small amount of Ni_31_Si_12_ crystals for the samples heated up to 786 K. With increasing annealing temperature to 852 K, the volume fraction of Ni_31_Si_12_ crystals slightly increased while the positions of the diffraction peaks corresponding to the BCC phase slightly shifted to lower diffraction angles. Meanwhile, some FCC crystals appeared, whose positions of the diffraction peaks remained almost constant during heating (c.f. the dot-dashed line in Figure 1a). Besides the BCC, FCC and Ni_31_Si_12_ crystalline phases, other crystals also precipitated in the amorphous matrix. As shown in Figures S1 and S2a in the Supplementary Materials, the crystalline diffraction peaks corresponding to these unknown phases probably came from Fe_2_B, Co_2_B, Ni_2_B, or the mixture of them but not the Laves phases. As we know, Laves phases are intermetallic compounds that have a stoichiometry of AB_2_ and are formed when the atomic size ratio is between 1.05 and 1.67 [40]. In our case, the atomic sizes of A (i.e., element B) and B (i.e., element Fe, Co, or Ni) are far less than 1, further eliminating the existence of Laves phases. Furthermore, as shown in Figure 1c and Figure S2b, the diffraction peak related with the crystallographic plane (111) of the FCC crystals, which exhibited a splitting behavior for the samples annealed at high temperatures, which was also observed in other diffraction peaks (the rectangle in Figure 1a). Furthermore, more XRD measurements were repeated, which were well consistent with each other (Figure 1 and Figure S2). These facts implied the possible formation of two types of FCC phases.

**Figure 2:**
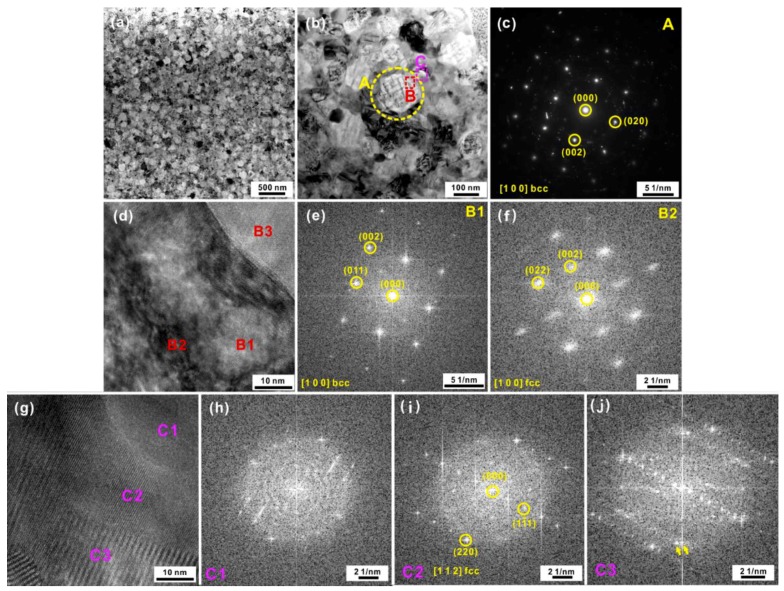
(**a**) Characteristic bright-field TEM image of the AR samples heated to 852 K and (**b**) their local microstructures, (**c**) the SAED pattern of the region A, (**d**) the HRTEM image of the region B, the FFT patterns of the domains (**e**) B1 and (**f**) B2, (**g**) the HRTEM image of the region C, the FFT patterns of the domains (**h**) C1, (**i**) C2, and (**j**) C3.

In order to further identify the observed crystalline phases, TEM and the selected area electron diffraction (SAED) measurements were conducted on the AR samples heated to 852 K since these samples contained all the crystalline phases observed during the whole crystallization process (Figure 1a). The investigations revealed that all of the crystallization products were nano-scale (Figure 2a) and several kinds of nano-scale crystalline phases precipitated (Figure 2b) in the amorphous matrix. As shown in Figure 2b, a large amount of bright particles with a size of 161 ± 28 nm (region marked by the dashed circle A), which consisted of nano-scale network-like precipitates, could be observed. According to the corresponding SAED pattern (Figure 2c), the dominant crystalline phase could be indexed as the BCC solid solution along the zone axis [100]. Moreover, other crystalline phases (regions B and C) within and around region A were also observed, while their structures and corresponding Fast Fourier Transformation (FFT) patterns are shown in Figure 2d–j. A characteristic high resolution TEM (HRTEM) image of the local microstructures of region B is shown in Figure 2d. A dark crystalline phase (region B2) alternately distributed within gray domains (B1), resulted in the formation of a network-like structure. According to the FFT patterns (Figure 2e,f), the B1 and B2 were identified as BCC and FCC solid solutions, respectively. Then it could be inferred that the FCC solid solution gradually precipitated within the primary metastable BCC solid solution matrix. Nearby the A regions which contained both BCC and FCC crystals, some amorphous-crystalline mixtures could be observed (B3), confirming the existence of the residual amorphous phase. Such amorphous-crystalline mixtures were also observed in region C (see the domain C1), whose local microstructure and FFT pattern are shown in Figure 2g,h, respectively. Besides the amorphous-crystalline mixture, FCC crystals (C2) could also be indexed based on the FFT pattern (Figure 2i). Among these FCC crystals, some twinned crystals (C3) could be observed, which was corroborated by their FFT patterns (Figure 2j).

**Figure 3:**
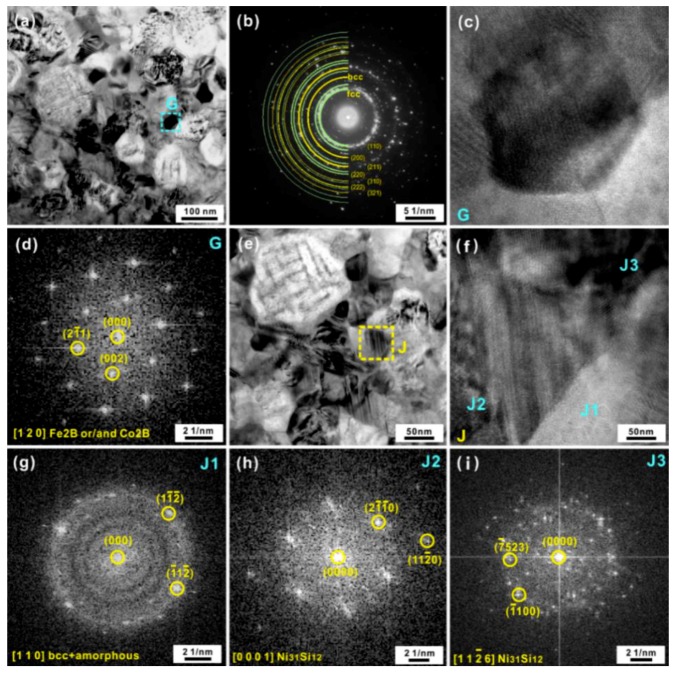
(**a**) Characteristic bright-field TEM image of other regions of the AR samples heated to 852 K and (**b**) the corresponding SAED pattern, (**c**) the HRTEM image of the region G and (**d**) the corresponding FFT patterns, (**e**) the local enlarged image of the region in Figure 2b, (**f**) the HRTEM image of the region J, the FFT patterns of the domains (**g**) J1, (**h**) J2, and (**i**) J3.

TEM and SAED investigations were also conducted in other regions (Figure 3a) in an attempt to further confirm the existence of other crystals besides the observed phases mentioned above. Based on the SAED patterns (Figure 3b), these regions are also governed by BCC and FCC crystals. Additionally, some dark nanoparticles embedded in the matrix could be found (region G in Figure 3a), whose HRTEM image is shown in Figure 3c. According to the SAED pattern (Figure 3d), these nanoparticles are identified as tetragonal Fe_2_B or Co_2_B along the zone axis [120]. Besides, HRTEM investigations (Figure 3e) were also conducted on other particles (Regions J). As shown in Figure 3f, the domain J1 within the region J (see Figure 3e) could be identified as a mixture of the amorphous and BCC phases (Figure 3g). Meanwhile, domains J2 and J3 within the region J were identified as Ni_31_Si_12_ intermetallic compounds (Figure 3h,i). Hence, it could be preliminarily referred that the FCC and BCC phases were the dominant crystallization products together with a few Fe_2_B, Co_2_B, and Ni_31_Si_12_ crystals in the amorphous matrix.

**Figure 4:**
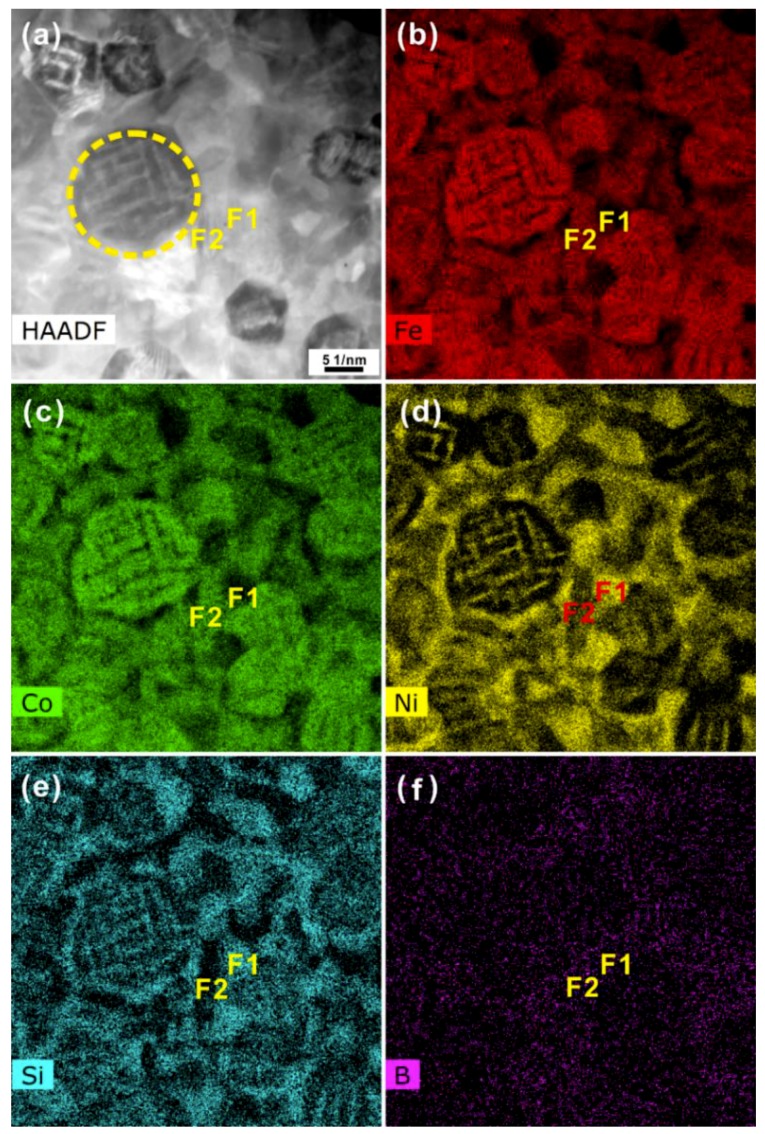
**Figure4.** (**a**) HAADF image of the AR sample heated to 852 K as well as its chemical distributions of elements (**b**) Fe, (**c**) Co, (**d**) Ni, (**e**) Si, and (**f**) B.

**Figure 5:**
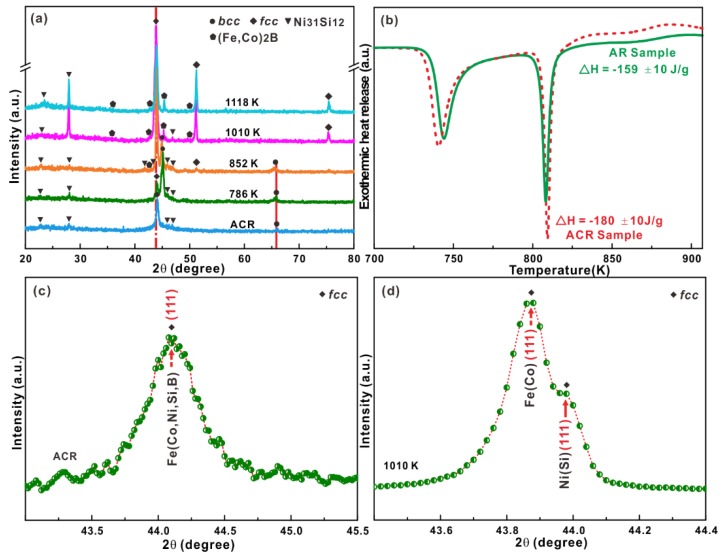
(**a**) XRD patterns of the melt-spun ACR ribbons and samples heated to different temperatures, (**b**) DSC curves of the AR and ACR samples, (**c**) the diffraction peak of the FCC phase along the zone axis (111) for the as-cast ACR sample (taken from Figure 5a), and (**d**) the diffraction peaks of two type of FCC phases along the zone axis (111) for the ACR sample heated to 1010 K (taken from Figure 5a).

Furthermore, the chemical distributions were also analyzed based on the HADDF-STEM together with EDX measurements (Figure 4a–f) performed on the local region shown in Figure 3a. These observation revealed that within the areas (see the circle in Figure 4a) containing BCC and FCC phases, the BCC crystals were rich in Fe, Co, and Si, while the FCC crystals were rich in Ni and Si (Figure 4b–f). Previous results have demonstrated that during crystallization of FeCoNi-based supercooled liquids, the metastable BCC supersaturated solid solution usually forms and the elements Co, Ni, Si, and B are easily able to dissolve into the BCC Fe phase [32,33,41,42,43]. For example, for Fe-Ni alloys with 10–35 at % Ni content, the nucleation of the metastable BCC phase becomes favorable by a nucleation trigger of a proper crystallographic structure and lattice parameters [42]. In our case, with the gradual precipitation of FCC crystals within the BCC crystals, the element Ni were gradually isolated from BCC crystals, leading to the formation of the newly-generated FCC Ni(Si) phase. In fact, M. Byshkin et al. has proven that the element Ni is easy to segregate from the Fe-rich BCC phases, resulting in the formation of the FCC phase [44]. The dissolution of a large amount of atoms Si in FCC Ni crystals would induce a large lattice distortion, possibly leading to the formation of nano twinning. When the content of the Si in Ni is larger than its solubility in Ni, the Ni-Si intermetallic compounds could be induced. As shown in (Figure 4a–f), the domain F1 was only rich in Ni and Si, corresponding to the Ni_31_Si_12_ intermetallic compound. Only Fe, Co, and B could be found in domain F2, which could be ascribed to be a mixture of both Fe_2_B and Co_2_B intermetallic compounds. Combining with the HRTEM and SAED investigations, the phase transformation from the amorphous phase → BCC Fe(Co, Ni, Si, B) + Ni_31_Si_12_ + amorphous phase → BCC Fe(Co) + FCC Ni(Si) + (Fe,Co)_2_B + Ni_31_Si_12_ + a little amorphous phase could be observed during crystallization at moderate temperatures for the AR sample. Upon further heating to 1010 K, no obvious diffraction peaks corresponding to the BCC phase could be observed, implying that the BCC solid solution disappeared at high temperatures because of its metastable nature. With heating to 1118 K and 1229 K, respectively, the positions of the diffraction peaks of the FCC phase remained almost constant (see the dot-dashed line in Figure 1a). This was in contrast to the change in the diffraction reflections of the BCC phase upon heating, confirming that the FCC phase was stable upon full crystallization. As we know, the polymorphic transformation from the FCC to BCC phase usually occurs at high temperatures for Fe-based, FeCo-based, and FeNi-based alloys during cooling [42,44]. As a result, the low-temperature metastable BCC Fe(Co) phase gradually transforms into the FCC Fe(Co) phase upon heating to high temperatures. Meanwhile, it is obviously seen that the content of Ni_31_Si_12_ precipitates increases according to their corresponding diffraction peaks (see the dotted line in Figure 5a). Thus, it can be concluded that the final crystallization products at high temperatures are some FCC Fe(Co) and FCC Ni(Si) crystals together with a little (Fe,Co)_2_B and Ni_31_Si_12_ crystals for the AR sample.

### 3.2. Microstructural Evolution of the Amorphous-Crystalline High-Entropy Alloy during Heating

In order to further verify the polymorphic BCC to FCC phase transformation, the amorphous-crystalline ACR HEAs were fabricated by decreasing the wheel speed to 11.52 m/s during melt spinning. Their characteristic XRD pattern shown in Figure 5a revealed that only the FCC solid solution together with a little Ni_31_Si_12_ crystals in the amorphous matrix were visible for the melt-spun ACR ribbons. Based on the DSC curves (Figure 5b and Figure S3), the heat enthalpy (Δ*H* = −180 ± 10 J/g) released during crystallization of the ACR samples was surprisingly larger than that (Δ*H* = −159 ± 10 J/g) of the AR samples. As a rule of thumb, fully amorphous alloys usually release a larger heat enthalpy in unit than their amorphous-crystalline counterparts. Therefore, the abnormal increase of the released heat enthalpy for the ACR samples during crystallization below *T_s_* should not originate from the crystallization of the amorphous matrix but from the other solid phase transformation. In our case, besides the amorphous matrix for the ACR samples, only pre-existing metastable FCC solid solution could be observed. Usually, when the cooling rate is suitable, only FCC solid solution can be observed in the amorphous matrix [32,33], which indicates that the primary FCC phase is one kind of supersaturated solid solution consisting of elements Fe, Co, Ni, Si, and B. As mentioned in Section 3.1, the metastable FCC solid solution tends to firstly form at high temperatures and then easily transform into the low-temperature metastable BCC solid solution. In our case, during rapid solidification, such a polymorphic phase transformation was suppressed to some content. As a result, a few metastable FCC crystals first precipitated from the melts, while the residual melts after the precipitation of FCC crystals were quenched into an amorphous state, leading to the formation of metallic glass composites. During rapid solidification, it is reasonable to assume that the FCC supersaturated solid solution would prefer to form without any decomposition. In fact, as shown in Figure 5c, the diffraction peak related to the crystallographic plane (111) of the FCC crystals did not show a splitting behavior compared with that observed in Section 3.1. Since the metastable BCC Fe phase is more stable than the primary metastable FCC Fe crystals at moderate temperatures [42,44], the pre-existing metastable FCC crystals in the amorphous matrix for the ACR samples gradually transformed into the metastable BCC phase after heating to 786 K, confirming the metastable nature of the primary FCC phase at room temperature and the abnormal increase of the released heat enthalpy for the ACR samples during crystallization below *T_s_*. However, upon increasing to 852 K, the newly-generated metastable BCC phase tended to transform back to two types of stable FCC Fe(Co) and FCC Ni(Si) phases accompanied by the formation of (Fe,Co)_2_B and Ni_31_Si_12_ crystals in the ACR samples. As shown in Figure 5d, the diffraction peak related to the crystallographic plane (111) of the FCC crystals indeed displayed splitting behaviors, further confirming our speculation. Such a transformation was similar with the crystallization behaviors of the AR sample upon heating. When heating to 1010 K, no BCC phase could be found but only FCC, (Fe,Co)_2_B, and Ni_31_Si_12_ crystals. In the past few decades, it has been shown that primary precipitates from melts during (rapid) solidification are usually different from the primary crystallization products of metallic glasses during heating [45,46,47]. These observations together with the findings presented in Section 3.1 further confirm that the FCC phase is metastable at moderate temperatures but stable at high temperatures. During rapid solidification (i.e., non-equilibrium solidification), the supersaturated FCC precipitates form in the undercooled melt/amorphous phase and severe solute redistributions between crystals and the amorphous matrix can be induced upon annealing at moderate temperatures. As shown in Figure 5a, the positions of the diffraction peaks related to the primary FCC phase at moderate temperatures and shifted to lower diffraction angles (the dot-dashed line). Meanwhile, the BCC phase gradually appeared while the positions of the diffraction peaks also shifted to lower diffraction angles. Upon heating at high temperatures (i.e., 1010 K and 1118 K), the newly-generated BCC intermediate phase became unstable and gradually transformed back into two types of FCC phases (i.e., FCC Fe(Co) and Ni(Si)). As a result, a metastable FCC → metastable FCC → stable FCC polymorphic phase transformation occurred for the amorphous-crystalline ACR samples upon heating.

### 3.3. Temperature Dependence of Magnetic Properties

The variation in the magnetic properties with temperature can act as an indicator to point out the phase transformation and microstructural evolutions occurring in the melt-spun samples upon heating. Hence, the variations of the saturation magnetization (*B_s_*) and the coercivity (*H_c_*) for the as-spun AR and ACR samples as well as the corresponding heat-treated states were measured by the VSM and *B*–*H* devices, respectively. As listed in Table 1, the melt-spun AR and ACR ribbons exhibited good soft magnetic properties, i.e., high *B_s_* and low *H_c_*. The as-spun AR ribbons had a *B_s_* of 1.005 ± 0.010 T and an *H_c_* of 5.3 ± 0.3 A/m. In case of the partially crystalline melt-spun ACR ribbons, where an amorphous phase coexists with the metastable nanocrystalline FCC Fe(Co,Ni,Si,B) supersaturated solid solution together with some Ni_31_Si_12_ crystals, *B_s_* slightly increased to 1.028 ± 0.010 T while *H_c_* reached 13.4 ± 3.2 A/m. As we know, the ferromagnetic state of FCC Co and Ni phases are relatively stronger than the antiferromagnetic state of the pure FCC Fe phase [48,49]. Besides, the FCC Fe(Co) and FCC Fe(Ni) phase also exhibit a ferromagnetic behavior when the contents of Co and Ni are well controlled [48,49]. In fact, the FCC Fe-Ni-Co alloys are also ferromagnetic [50]. Therefore, it is reasonable to believe that the primary FCC Fe(Co,Ni,Si,B) crystals also exhibit ferromagnetic behavior. Additionally, it has been demonstrated that the density of amorphous alloys upon crystallization increases up to ~0.6% [51], which can lead to the slight change in *B_s_* for the ACR ribbons. When melt-spun ribbons were heated to 786 K, the *B_s_* of the AR and ACR ribbons increased to 1.108 ± 0.020 T and 1.068 ± 0.020 T, respectively. According to the XRD and TEM results (Figure 1, Figure 2, Figure 3, Figure 4 and Figure 5) and previous reports [32,33,41,42,43], the main crystallization products for the AR samples were the metastable BCC Fe(Co,Ni,Si,B) crystals in nano-scale, while the precipitation of a mixture of the metastable BCC Fe(Co) and FCC Ni(Si) crystals governed the partial crystallization for the ACR samples. It is well known that Fe-based nanocrystalline-amorphous alloys with precipitated nanocrystalline BCC-Fe grains in the amorphous matrix after optimized annealing exhibit excellent soft magnetic properties [50,52,53,54]. Therefore, for the present samples, the *B_s_* could be further improved for both types of ribbons through the precipitation of nano-scale BCC crystals. For the ACR samples, the presence of few ferromagnetic FCC Ni(Si) crystals were believed to cause a lower *B_s_* compared with the AR samples due to the smaller atomic magnetic moment of the FCC Ni phase (i.e., 1.26 *μ_B_*) than BCC (FeCo) phase (i.e., 2.0–2.4 *μ_B_* depending on the Co content) [55,56,57]. On the other hand, the *H_c_* for both the AR and ACR samples strongly increased to more than 200 A/m. It has been demonstrated that Fe-based amorphous alloys usually exhibit a smaller *H_c_* than their crystalline counterparts due to their densely packed glassy structure and the disappearance of the magneto-crystalline anisotropy, while the *H_c_* usually increases with non-magnetic or weakly-magnetic crystalline phases precipitating in the amorphous matrix [52,53,54,58,59]. Then the gradual disappearance of the amorphous phase would induce the increase of *H_c_* upon crystallization.

When the samples are heated to 852 K, the dominant crystallization products for both the AR and ACR ribbons are a mixture of nano-scale FCC and BCC crystals together with some intermetallic compounds without any amorphous phase. Hence, the *B_s_* for both samples heated to 852 K was comparable (i.e., 1.107 ± 0.020 T and 1.095 ± 0.023 T, respectively). Additionally, the *H_c_* of the AR samples annealed at 852 K slightly increased while the ACR samples annealed at 852 K decreased. The difference may result from slightly different ratios of both FCC and BCC crystals in the AR and ACR samples. Finally, when both types of ribbons were heated to 1010 K, the precipitated phases became similar, leading to a similar *B_s_*. As we know, the *H_c_* strongly depends on the magneto-crystalline anisotropy energy [60]. At 1010 K, the content of hard magnetic (Fe,Co)_2_B crystals becomes dominant for both samples according to their intensities of the x-ray diffraction peaks (Figure 1a and Figure 5a). It has been reported that the maximum magneto-crystalline anisotropy energy of (Fe,Co)_2_B phase can reach 15000 kJ/m^3^ [61], which is larger than that of Fe(Co) phases [62]. Besides, since the polymorphic transformation is entirely finished at 1010 K, the further increase of the intensities of the x-ray diffraction peaks related with FCC, (Fe,Co)_2_B, and Ni_31_Si_12_ crystals should be attributed to the grain growth and coarsening, which can also induce the increase of *H_c_* for the AR and ACR samples annealed at 1010 K.

## 4. Conclusions

In this work, the correlation between the microstructural features and magnetic properties of the high-entropy Fe_26.7_Co_26.7_Ni_26.7_Si_8.9_B_11.0_ amorphous ribbons and (partially) crystalline ribbons were investigated. Upon annealing fully amorphous AR ribbons, the primary BCC Fe(Co,Ni,Si,B) crystals together with some Ni_31_Si_12_ intermetallic compounds precipitated in the amorphous matrix. With further increasing temperature, the FCC Ni(Si) crystals gradually precipitated within the primary BCC crystals, and consequently the BCC crystals were rich in Fe and Co. Subsequently, the BCC Fe(Co) phase gradually transformed into FCC Fe(Co) phases accompanied by further precipitation of (Fe,Co)_2_B and Ni_31_Si_12_ crystals. On the other hand, when the cooling rate during rapid solidification was reduced, the primarily solidified FCC Fe(Co,Ni,Si,B) crystals coexisted with the amorphous matrix, leading to the formation of high-entropy crystalline-amorphous ribbons. When the crystalline-amorphous ACR ribbons were gradually heated to 786 K and then 1010 K, the metastable FCC Fe(Co,Ni,Si,B) → metastable BCC Fe(Co,Ni,Si,B) → FCC Fe(Co) + FCC Ni(Si) polymorphic transformation occurred. With increasing temperature, the *B_s_* of the AR and ACR gradually increased and then decreased, while the *H_c_* rose except the sudden drop at 852 K for the ACR sample, which strongly depended on the complicated and gradual microstructural transition upon heating fully amorphous or crystalline-amorphous ribbons.

## Figures and Tables

**Table 1:** *B_s_* and *H_c_* of the as-quenched AR and ACR ribbons and samples heated at different temperatures.

Samples	Temperatures (K)	*B_s_* (T)	*H_c_* (A/m)
AR	273 (As-cast)	1.005 ± 0.010	5.3 ± 0.3
	786	1.108 ± 0.020	205.8 ± 16.8
	852	1.107 ± 0.020	207.8 ± 9.8
	1010	0.973 ± 0.002	225.4 ± 14.9
ACR	273 (As-cast)	1.028 ± 0.010	13.4 ± 3.2
	786	1.068 ± 0.020	235.0 ± 3.9
	852	1.095 ± 0.023	156.2 ± 15.2
	1010	0.970 ± 0.020	288.3 ± 10.0

## Supplementary Materials

The following are available online at http://www.mdpi.com/1996-1944/12/4/590/s1, Figure S1: XRD of the melt-spun AR ribbons heated to 1118 K and the Powder Diffraction File (PDF) cards of Fe_2_B, Co_2_B, Ni_2_B, the cubic MgCu_2_ (C15), hexagonal MgZn_2_ (C14), and hexagonal MgNi_2_ (C36) Laves phases, respectively, Figure S2: (a) XRD curves of the melt-spun AR ribbons heated to different temperatures at a heating and cooling rate of 20 K/min, and (b) the local enlarged diffraction peaks of two type of FCC phases along the zone axis (111) for the ACR sample heated to 1010 K, Figure S3: DSC curves of the melt-spun AR and ACR ribbons at a heating and cooling rate of 20 K/min.
