# Supplementary material for: Polymorphic Transformation and Magnetic Properties of Rapidly Solidified Fe26.7Co26.7Ni26.7Si8.9B11.0 High-Entropy Alloys
Source: Materials (Basel). 2019 Feb 15;12(4):590. doi: 10.3390/ma12040590 (PMC6416621; doi:10.3390/ma12040590)
Supplement: Supplementary file 1 [file materials-12-00590-s001.zip › materials-428163-SI.pdf]

materials

# Supplementary Materials: Polymorphic Transformation and Magnetic Properties of Rapidly Solidified $Fe_{26.7}Co_{26.7}Ni_{26.7}Si_{8.9}B_{11.0}$ High Entropy Alloys

**Zequn Zhang, Kaikai Song, Ran Li, Qisen Xue, Shuang Wu, Delong Yan, Xuelian Li, Bo Song, Baran Sarac, Jeong Tae Kim, Parthiban Ramasamy, Li Wang and Jürgen Eckert**

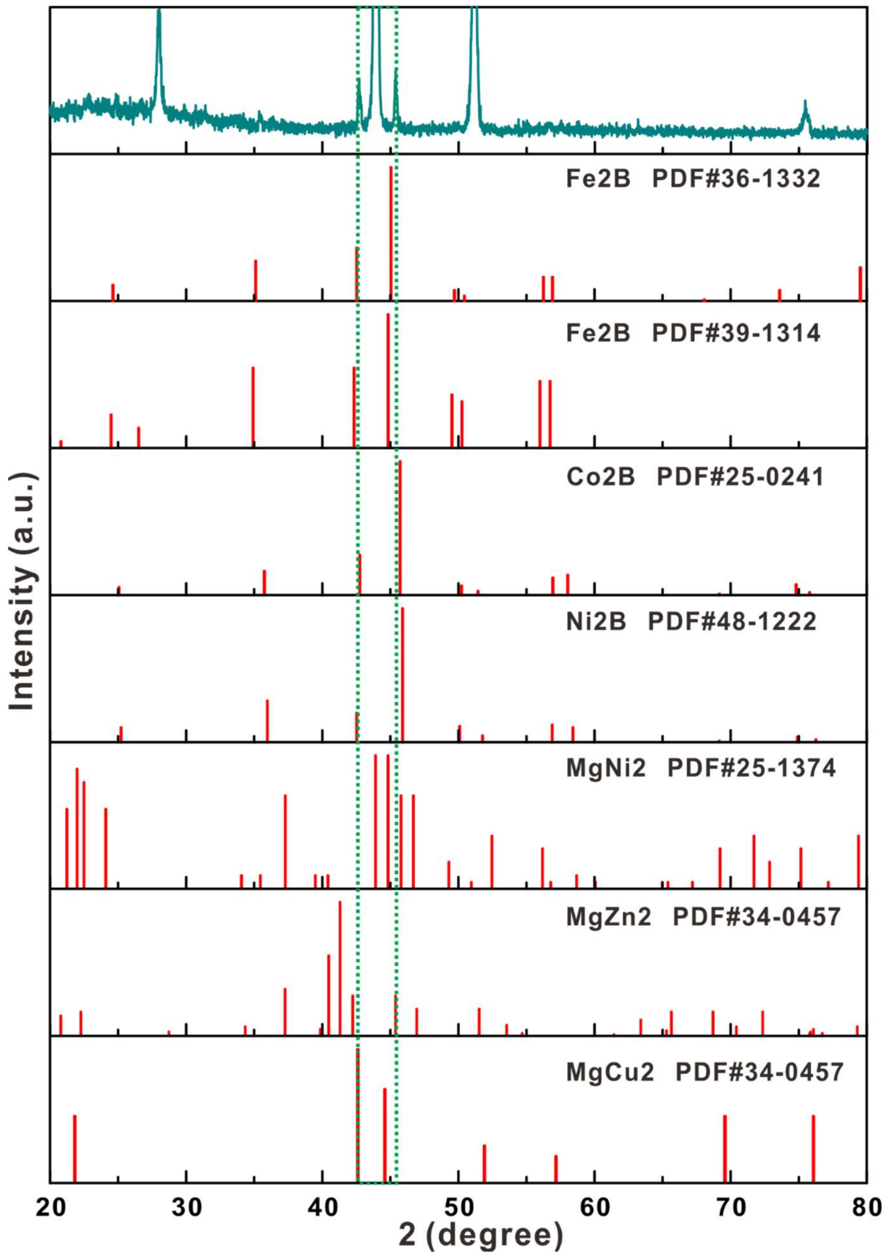


**Figure S1.** XRD of the melt-spun AR ribbons heated to 1118 K and the Powder Diffraction File (PDF) cards of $Fe_2B$, $Co_2B$, $Ni_2B$, the cubic $MgCu_2$ (C15), hexagonal $MgZn_2$ (C14), and hexagonal $MgNi_2$ (C36) Laves phases, respectively [1-4].

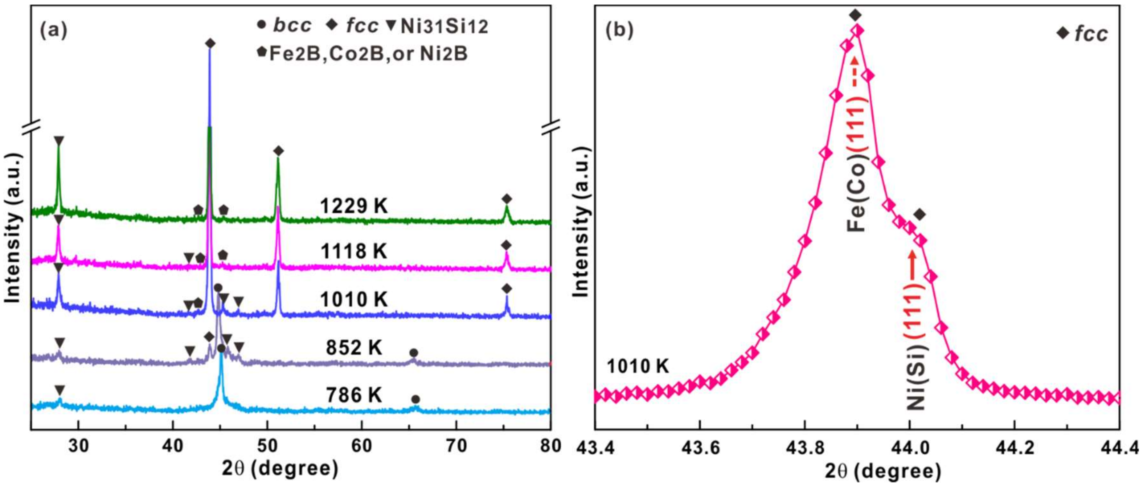


**Figure S2.** (**a**) XRD curves of the melt-spun AR ribbons hated to different temperatures at a heating and cooling rate of 20 K/min, and (**b**) the local enlarged the diffraction peaks of two type of *fcc* phases along the zone axis (111) for the ACR sample heated to 1010 K.

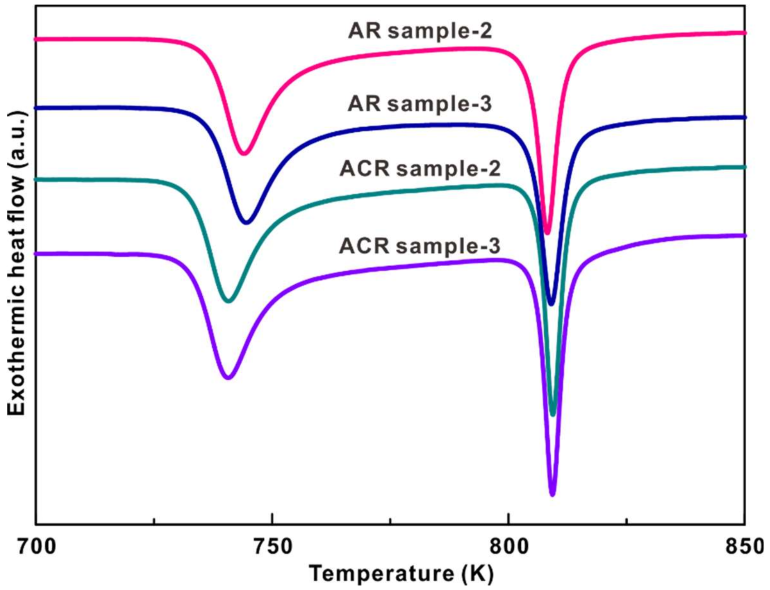


**Figure S3.** DSC curves of the melt-spun AR and ACR ribbons at a heating and cooling rate of 20 K/min.

## References

1. Havinga, E.E.; Damsma, H.; Hokkeling, P. Compounds and pseudo-binary alloys with the $CuAl_2$(C16)-type structure I. Preparation and X-ray results. *J. Less-Common Met*. **1972**, 27, 169-186.
2. Hanawalt, J.; Rinn, H.; Frevel, L. Chemical Analysis by X-Ray Diffraction. *Anal. Chem.* **1938**, 10, 457.
3. Laves, F.; Witte, H. Crystal structure of $MgNi_2$ and its relation to the types $MgCu_2$ and $MgZn_2$. *Metallwirtschaft* **1935**, 14, 645.
4. http://www.icdd.com/

© 2019 by the authors. Submitted for possible open access publication under the terms and conditions of the Creative Commons Attribution (CC BY) license (http://creativecommons.org/licenses/by/4.0/).
